# Life on Magnet: Long-Term Exposure of Moderate Static Magnetic Fields on the Lifespan and Healthspan of Mice

**DOI:** 10.3390/antiox12010108

**Published:** 2022-12-31

**Authors:** Yixiang Fan, Xin Yu, Biao Yu, Xinmiao Ji, Xiaofei Tian, Chao Song, Xin Zhang

**Affiliations:** 1 High Magnetic Field Laboratory, Hefei Institutes of Physical Science, Chinese Academy of Sciences, Hefei 230031, China; 2 Science Island Branch of Graduate School, University of Science and Technology of China, Hefei 230036, China; 3 Institutes of Physical Science and Information Technology, Anhui University, Hefei 230601, China; 4 International Magnetobiology Frontier Research Center, Science Island, Hefei 230036, China

**Keywords:** static magnetic field, senescence, oxidative stress, ageing, PC12 cells

## Abstract

All living organisms on the Earth live and evolve in the presence of the weak geomagnetic field, a quasi-uniform static magnetic field (SMF). In the meantime, although the effects of moderate and high SMFs have been investigated on multiple aspects of a living organism, a long-term SMF exposure of more than 1 year has never been reported. Here, we investigated the influence of a moderate SMF (70–220 mT head-to-toe) long-term continuous exposure (1.7 years) to two different SMF directions on healthy male C57BL/6 mice. We found that not only was the lifespan of the mice prolonged, but their healthspan was also improved. The elevated plus maze test and open field test show that SMFs could significantly improve the exploratory and locomotive activities of the aged mice. The Morris water maze test shows that SMFs could improve their spatial learning ability and spatial memory. Tissue examinations reveal that SMFs have an ameliorative effect on oxidative stress in the brain of aged mice, which was reinforced by the cellular assays, showing that SMFs could protect the PC12 cells from D-gal-induced senescence by increasing superoxide dismutase, catalase, and reducing the malonaldehyde levels. Therefore, our data show that the 1.7-year SMF exposure can improve both the lifespan and healthspan of naturally aged mice due to reduced oxidative stress, which indicates that SMFs have the potential to be used as an adjuvant physical therapy to reduce the ageing-induced health risks to benefit animals, and even humans.

## 1. Introduction

The life expectancy of human beings has been significantly increased in the past century. Ageing can be characterized by the progressive loss of physiological integrity as a result of decreased organismal function, which can give rise to many common diseases in older adults, such as diabetes, cancer, cardiovascular disease and neurodegenerative disorders [1,2]. Therefore, the healthspan is also the focus of ageing research as well as the lifespan. In fact, a change in lifestyle or using some pharmacological interventions has been shown to be able to delay the onset of multiple age-related diseases and improve the lifespan and healthspan [3,4,5]. Moreover, geroprotectors, a new class of drugs that target ageing, have been developed in recent years [6]. For example, rapamycin has been confirmed to be able to extend the lifespan and improve the health span of different animal models [7,8,9], and metformin can also mitigate ageing [10]. However, the clinical uses of rapamycin have been reported to induce hyperglycemia, hyperlipidemia, kidney toxicity, impaired wound healing, lowered blood platelet numbers, and immunosuppression [11,12]. Metformin was reported to inhibit the increase in the sensitivity to systemic insulin and skeletal muscle mitochondrial respiration during aerobic exercise training in older adults [13]. Therefore, it is particularly important to develop a safe and effective method for extending the lifespan as well as boosting the resilience to multiple age-related diseases.

Moderate SMFs have been indicated to be a potential non-invasive physical method to improve some pathological conditions, such as cancer [14]. Recently, Song et al. showed that a 10 mT SMF could improve the longevity of C. elegans [15]. In the meantime, although the safety guideline for the public has been set by the International Commission on Non-Ionizing Radiation Protection (ICNIRP) to be 0.4 T [16], and our group has reported that an exposure to as high as 33.0 T SMF for 1 h did not have obvious harmful effects, and could even reduce the anxiety levels and improve the sociality and spatial memory of normal C57BL/6 mice [17,18], the exact effects of a long-term SMF exposure of more than one year on living organisms are still unclear. The longest SMF exposure experiment so far was reported in 1986 by Bellossi et al., who found that a 600 or 800 mT SMF exposure for one year could prolong the survival of female spontaneous lymphocytic leukemia mice [19]. However, we have recently showed that some specific types of moderate SMF treatment for weeks could generate detrimental effects on binge drinking mice, which significantly shortened their survival time [20]. Therefore, the exact consequence of an SMF exposure on living organisms are correlated with the magnetic flux density, treatment time, as well as the physiological conditions of the living organisms themselves.

The purpose of this study is to conduct a 1.7-year SMF exposure experiment to investigate the influence of a long-term exposure on the lifespan and healthspan of naturally aged mice. Since SMFs of different directions have also been shown to have differential effects [21,22,23,24], here we also set up both the vertically upward and downward direction SMFs for comparison.

## 2. Materials and Methods

### 2.1. Magnetic Field Exposure Device

**Figure 1:**
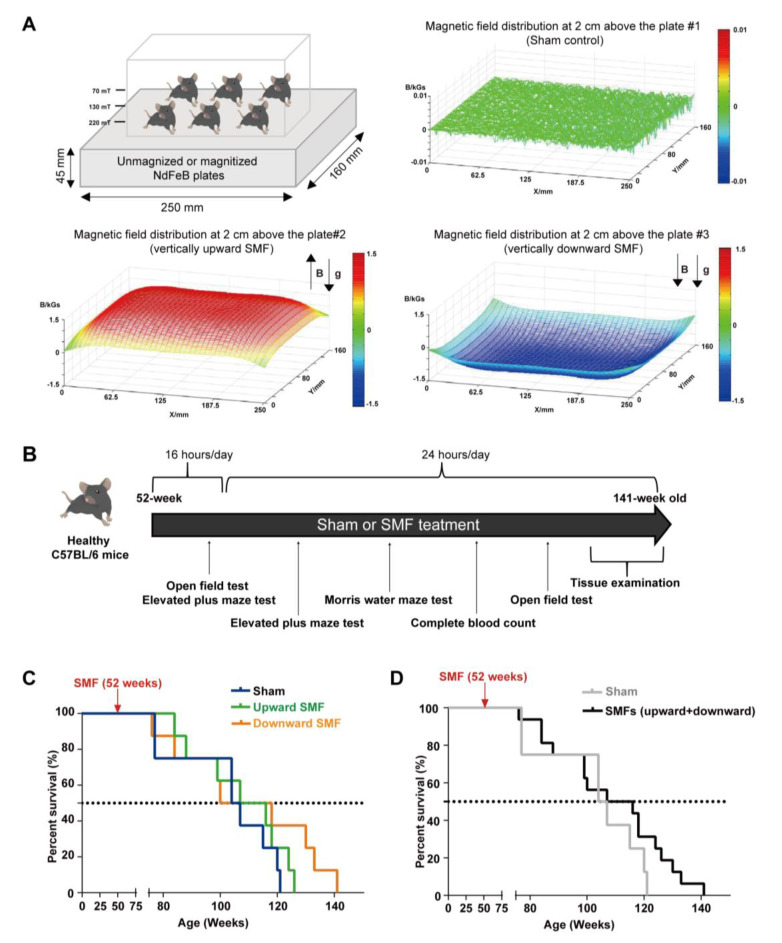
Moderate quasi–uniform SMF long–term exposure prolongs mice lifespan. (**A**) A diagram of the mice cage placed on sham control vs. two different magnetic plates that provide vertically upward and downward direction SMFs. Magnetic flux densities were scanned at 2 cm above the plates; (**B**) experimental design flow chart; (**C**,**D**) the survival rate of naturally aged mice. The survival rate recorded for naturally aged mice in vertically upward or downward SMF exposure groups was analyzed separately (**C**) or grouped together as SMFs (**D**). n = 8 mice for the sham control, upward SMF or downward SMF group in the beginning of the experiment.

The devices for animal experiments are shown in Figure 1A. The magnetic plates for the animal experiments are composed of 12 magnetized (to provide upward or downward SMFs) or unmagnetized (for the sham control) neodymium magnets, which was described previously [21]. The distribution of SMFs at 2 cm above the magnetic plates were measured by a magnet analyzer (FE-2100RD; Hunan Forever Elegance Technology, China). 

The devices for the cellular experiments, which are also made from magnetized or unmagnetized neodymium magnets, were placed in a 37 °C CO_2_ cell incubator. The sham control was placed in the same incubator as those in the SMF groups, but far from the SMF group. The distribution of SMFs at 1 mm above the magnets were measured by a magnet analyzer.

### 2.2. Animal Model

Twenty-four male C57BL/6 mice at 28 weeks old were obtained from the Nanjing GemPharmatech Co., Ltd. (Nanjing, China), which were randomly divided into three groups and routinely fed and kept in an air-conditioned room (21 °C to 25 °C) with 50% to 60% humidity, a light and dark cycle of 12 h. The mice were normally fed until they were 52 weeks old before the application of a sham or SMF treatment. The caring and handling of the mice were carried out in terms of the rules of the Care and Use of Laboratory Animals (NIH Publications No. 8023, revised 1978). The health conditions of the mice were checked every day and their death time was recorded. 

### 2.3. Behavioral Tests

Three different behavioral tests were performed in this study. To minimize the experimental variations, we performed all of the behavioral tests during the hours of 09:00–19:00.

#### 2.3.1. Open Field Test

An open field test (OFT) was used to measure the mice’s locomotor, exploratory, and anxiety-like behavior at 56 weeks old (after 4 weeks of SMF exposure) or 107 weeks old (after 55 weeks of SMF exposure), respectively. The apparatus (SA215, SANS, China) consisted of white plastic boards (1000 × 1000 × 400 mm) divided into four identical square areas that can accommodate four mice simultaneously. There is a central area (300 × 300 mm) and a peripheral area for each enclosed square area. The mice were placed at the center of the square area individually and were allowed to move freely for 5 min, and their movement was monitored by a video camera mounted above the apparatus. The movement parameters, including the total average velocity, total traveled distance, and time in the center area, were recorded and analyzed automatically by the ANY-Maze Video Tracking System (Stoelting, IL, USA). After each test, the apparatus was thoroughly cleaned with 75% ethanol.

#### 2.3.2. Elevated plus Maze Test

An elevated plus maze test (EPM) was conducted to evaluate the anxiety-like behavior at of the 56-week-old (after 4 weeks of SMF exposure) or 61-week-old (after 9 weeks of SMF exposure) mice, respectively. The cruciform apparatus (SA211, SANS, China) was made up of two oppositely positioned open arms (300 × 50 × 5 mm), two closed arms (300 × 50 × 150 mm), and a center area (50 × 50 mm). When the experiment started, the mice were placed in the center area. The time in the open arms, the total traveled distance, and the total average velocity were recorded for 5 min by a video camera mounted above the maze and analyzed by the ANY-Maze Video Tracking System (Stoelting, IL, USA). After each test, the apparatus was thoroughly cleaned with 75% ethanol.

#### 2.3.3. Morris Water Maze

The Morris water maze (MWM) test was performed to assess the spatial learning and memory of the 84-week-old mice (after 32 weeks of SMF exposure). The experiment was performed as previously described [25]. The apparatus (SA201, SANS, China) included a round pool (120 cm diameter and 40 cm height) filled with opaque water (with non-toxic white paint added, (25 ± 1 °C)). The pool was surrounded by a thick curtain to minimize the external noise and cues. A removable circular platform (of an 8 cm diameter and 20 cm height) was located in the maze for the mice to escape from the water. 

The pool was divided equally into 4 hypothetical quadrants: east (E), west (W), north (N), and south (S). The hidden platform was immersed 1.5 cm under the water surface in the SW quadrant, and the mice were released gently from four different positions (N, SE, E, and NW), who actively searched for escape pathways until they found the hidden platform. The mice were trained for four trials per day for four consecutive days. Each experiment was conducted for a maximum length of 60s. At the end of the trial, the mice that failed to find the hidden platform within 60s were guided and allowed to stay on the platform for 15s. The swimming pathway, their swimming speed, total distance, and the time taken to reach the platform (escape latency) were recorded by a video camera mounted above the maze and analyzed by the ANY-Maze Video Tracking System (Stoelting, IL, USA). The searching strategies were also analyzed. On the 5th day, the platform was removed from the pool, and the mice were released individually from the NE points. The animals were allowed to swim freely for 60s. A video camera mounted above the pool and the automated ANY-Maze Video Tracking System recorded the dwell time of the mice in a target quadrant and platform crossing.

### 2.4. Complete Blood Count Analysis

Blood samples were collected from the mice when they were 95 weeks old (after 43 weeks of SMF exposure) using a blood lancet. Then, 200 μL of the blood samples were transferred to a tube with 0.15% (M/V) EDTA-K_2_-2H_2_O anticoagulant and subjected to a complete blood count by an automatic hematology analyzer (Sysmex, Japan). We measured the red blood cell, hematocrit, hemoglobin, platelet, white blood cell, neutrophil, eosinophil, monocyte, lymphocyte, mean corpuscular volume, mean platelet volume, plateletcrit, mean corpuscular hemoglobin, mean corpuscular hemoglobin concentration, and platelet distribution width.

### 2.5. Sample Collection

After their death, the mice brain tissues were taken, weighed, and then homogenized in phosphate buffer on ice using a tissue grinder. After centrifugation at 3000g for 10 min at 4 °C, the supernatant was used for subsequent experiments.

### 2.6. Cell Culture and Cell Counting Kit-Eight Assays (CCK-8)

The differentiated rat pheochromocytoma PC12 cell line was obtained from the American Type Culture Collection (ATCC, CRL-1721) and maintained in DMEM containing 10% FBS and 1% penicillin/streptomycin. The cells were incubated at 37 °C with 5% CO_2_ (Thermo Scientific, CO_2_ incubator). 

To determine the optimal incubation concentration for D-Galactose (D-gal) (Sigma, USA), a cell counting kit-8 (CCK-8) assay (Beyotime Biotechnology, China) was used to measure the cell viability. The differentiated PC12 cells (3 × 10^3^ cells/well) were seeded in 96-well plates for 24 h. Then, the cells were washed and 0, 2.5, 5, 10, 20, 40, or 100 mg/mL of D-gal were added. After 48 h, 10 μL of CCK-8 solution were added to each of the 96-well plates, and the mixture was incubated for an additional 45 min at 37 °C. The absorbance was recorded at 450 nm using a Microplate Reader (Bio-Rad, USA). 

### 2.7. Senescence-Associated β-Galactosidase (SA-β-Gal) Activity Assay and Staining

The PC12 cells were seeded into a 3.5 cm plate (8 × 10^4^ cells/mL). After the attachment, the medium was supplemented with 20 mg/mL of D-gal, with a sham, upward, or downward SMF treatment for 48 h. An SA-β-gal activity assay kit (Sinobestbio, China) was used and all of the steps were carried out according to the manufacturer’s instructions. After being treated as was aforementioned, the SA-β-gal staining kit (Beyotime Biotechnology, China) was used to assess the cellular senescence.

### 2.8. The Measurement of Oxidative Stress Biomarkers

The total antioxidant capacity (T-AOC), superoxide dismutase (SOD), catalase (CAT) activities, glutathione (GSH), lipid hydroperoxide (LPO), nitric oxide (NO), and malonaldehyde (MDA) contents were measured using commercial kits (Beyotime Biotechnology, China) according to the manufacturer’s instructions.

### 2.9. Statistical Analysis

The data in Table 1 are expressed as the means ± standard deviation (SD) and other data are expressed at the means ± standard error of the mean (SEM). Graphpad prism 8 was used to analyze the data using Student’s *t*-test for the two groups. In all analyses, *p* < 0.05 were considered statistically significant. * *p* < 0.05, ** *p* < 0.01, *** *p* < 0.001, and **** *p* < 0.0001. Most of the experiments were repeated by two independent researchers or analyzed in a blind way to reduce the potential experimenter bias. 

## 3. Results

### 3.1. SMFs Increased the Lifespan of Mice

To determine the SMF-induced physiological effect on normal mice, we exposed 52-week-old male C57BL/6 mice to the sham control, vertically upward or downward SMF for 16 h per day for five weeks, and then 24 h per day, 7 days a week until their natural death (Figure 1A). Three different behavioral tests, including the open field test, elevated plus maze test, and the Morris water maze test, were used to evaluate their cognitive functions. A complete blood count was also performed to help assess their health status (Figure 1B).

We examined the effects of an SMF exposure on the lifespan of the mice. We found that SMFs prolonged both of the mice’s median and maximum lifespan (Figure 1C,D). Compared with the sham group (105.50 weeks), both the upward SMF (111.50 weeks) and downward SMF (109.00 weeks) increased the median lifespan of the mice (Figure 1C). Furthermore, the maximum lifespans in the upward SMF group (126.00 weeks) and downward SMF group (141.00 weeks) were both longer than that of the sham group (121.00 weeks) (Figure 1C). We also pooled the data from both the upward SMF and downward SMF together to assess the overall effects of SMFs. It is obvious that these moderate SMFs prolonged the lifespan of the naturally aged C57BL/6 mice (Figure 1D).

### 3.2. SMFs Reduced the Anxiety-Like Behavior and Enhanced Mice Locomotive and Exploratory Activities

**Figure 2:**
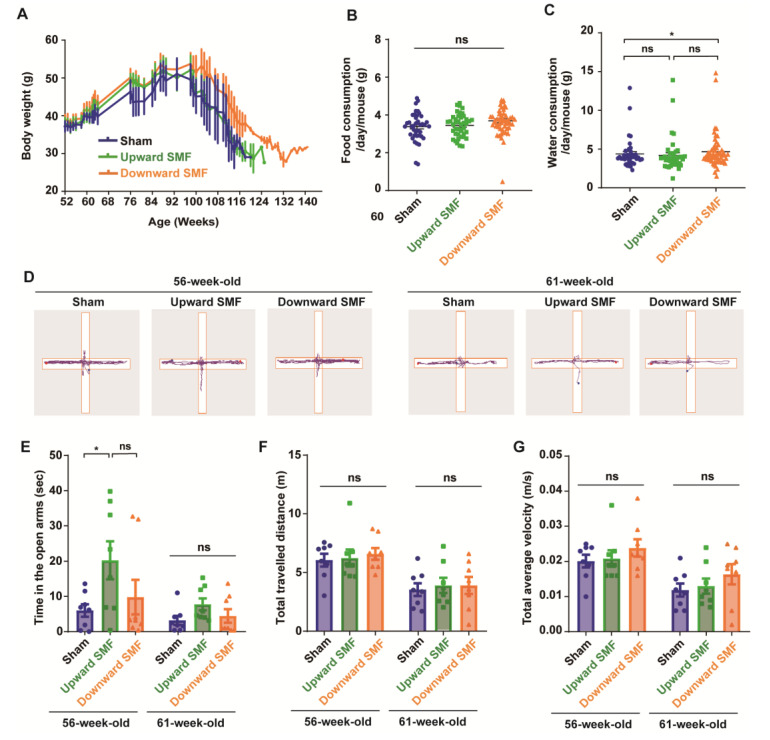
Elevated plus maze test shows that SMFs could improve the exploration activity of aged mice. (**A**) The mice body weight changes; (**B**) food consumption; (**C**) water consumption; (**D**) representative trajectories of mice in elevated plus maze test at 56 and 61 weeks old, respectively. The blue and red points show start and end points, respectively; (**E**) time in the open arms; (**F**) total traveled distance; (**G**) total average velocity. n=8 mice for the sham, upward SMF or downward SMF group in the beginning of the experiment. Values were expressed as means ± SEM. * *p* < 0.05 by Student’s *t*-test. ns, no significance.

In the meantime, to monitor the overall health status of the mice, we also examined their body weight, as well as their food and water consumption. Our results indicated that the body weight was slightly increased in the downward SMF group, but in general, the SMF-induced body weight changes were negligible (Figure 2A and Figure S1). In addition, the SMF treatment had no apparent effects on the food consumption of mice (Figure 2B and  Figure S1). The water consumption was slightly increased in the downward SMF group (Figure 2C). Moreover, we also measured the complete blood count at 95 weeks old (after 43 weeks of SMF exposure) and found that SMFs did not induce statistically significant differences (Table 1 and Figure S2).

To assess the effects of SMFs on the anxiety level of mice, the elevated plus maze (EPM) was performed on mice who were 56 or 61 weeks old. The mice naturally preferred to stay in the closed arm and occasionally explore the open arms (Figure 2D). Therefore, the time in the open arms is usually considered to be an indicator to reflect the exploratory activity and anti-anxiety level. It seems that the mice had a reduced time in the open arm, the total travelled distance, and velocity at 61 weeks of age compared to 56 weeks. However, it is interesting that the upward SMF exposure increased the time in the open arm by 237.39% (*p* < 0.05) and 141.25% (*p* = 0.05) at 56 and 61 weeks compared to the sham group (Figure 2E). In general, the SMFs treatment increased the time in the open arms, but with no statistical significance (Figure S3). In addition, the total traveled distance and total average velocity had no significant difference in the SMF treatment group compared to the sham group at 56 or 61 weeks old (Figure 2F,G and Figure S3). These results indicate that SMFs might have an anti-anxiety effect in mice.

**Figure 3:**
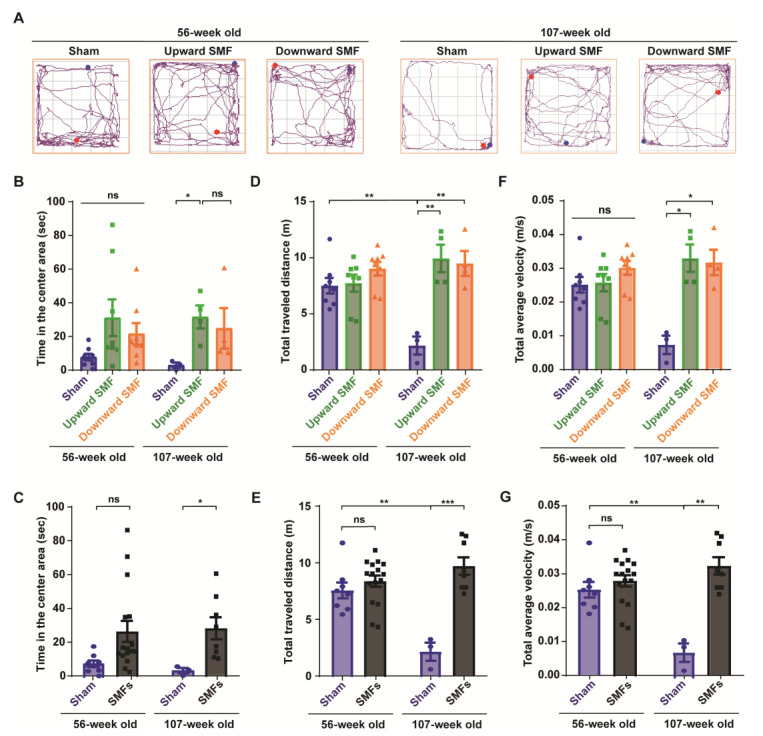
Open field test shows that SMFs could significantly improve the exploratory and locomotive activities of aged mice. (**A**) Representative trajectories of mice in the open field test at 56 and 107 weeks old. The blue and red points show start and end points, respectively; (**B**,**C**) time in the center area, (**D**,**E**) total traveled distance, (**F**,**G**) total average velocity were recorded and analyzed in a blinded way. Mice in the upward or downward SMF exposure groups were analyzed separately (**B**,**D**,**F**) or grouped together as SMF group (**C**,**E**,**G**). n = 8 mice for the sham, upward SMF or downward SMF group in the beginning of the experiment. Values were expressed as means ± SEM. * *p* < 0.05, ** *p* < 0.01, and *** *p* < 0.001 by Student’s *t*-test. ns, no significance.

To further evaluate the mental state of the mice, an open field test was performed to measure the locomotor activity of the mice, exploratory and anxiety-like behavior at 56 or 107 weeks old (Figure 3). The experiments were performed by one researcher, who recorded the travel path of each mouse by video, and the video was subject to a blind analysis by another researcher to reduce any potential experimenter bias (Figure 3A and Figure S4). The time in the center area is usually considered to be an indicator of the exploratory and anti-anxiety level, because the mice usually tend to stay in the around regions, especially at the corner (Figure S4). However, it was clear that the SMF-exposed mice spent more time in the center area than the sham group (Figure 3B,C and Figure S4). For example, the time in the center area in the upward SMF group increased by 302.10% (*p* = 0.05) and 1063.35% (*p* < 0.05) compared to the sham group at 56 and 107 weeks old, respectively. The time in the center area in the downward SMF group increased by 178.55% (*p* = 0.05) compared to the sham group at 56 weeks old (Figure 3B). As shown in Figure 3C, the time in the center area in the SMF treatment group increased by 245.97% (*p* = 0.05) and 862.64% (*p* < 0.05) compared to the sham group at 56 and 107 weeks old (Figure 3C). 

Moreover, in the open field test, the total traveled distance and total average velocity are also routinely used to reflect the locomotive activity of the mice. Different from the elevated plus test, in which the mice were only exposed to SMFs for 9 weeks at 61 weeks old, the total traveled distance and total average velocity in the SMF groups were significantly higher than in the sham group in the open field test, in which the mice were exposed to SMFs for 55 weeks at 107 weeks old (Figure 3D–G). For example, the total traveled distance in the upward and downward SMF group had increased by 357.85% (*p* < 0.01) and 337.27% (*p* < 0.01), respectively, compared to the sham group at 107 weeks old (Figure 3D). In general, the total traveled distance of the SMF treatment group increased by 347.56% (*p* < 0.001) compared to the sham group at 107 weeks old (Figure 3E). Furthermore, the total average velocity of the upward SMF group and downward SMF group mice increased by 350.20% (*p* < 0.05) and 333.15% (*p* < 0.05), respectively, compared to the sham group at 107 weeks old (Figure 3F). In general, the total average velocity of the SMFs treatment group increased by 341.48% (*p* < 0.01) compared to the sham group at 107 weeks old (Figure 3G). 

### 3.3. SMFs Improved Spatial Learning and Memory Ability

**Figure 4:**
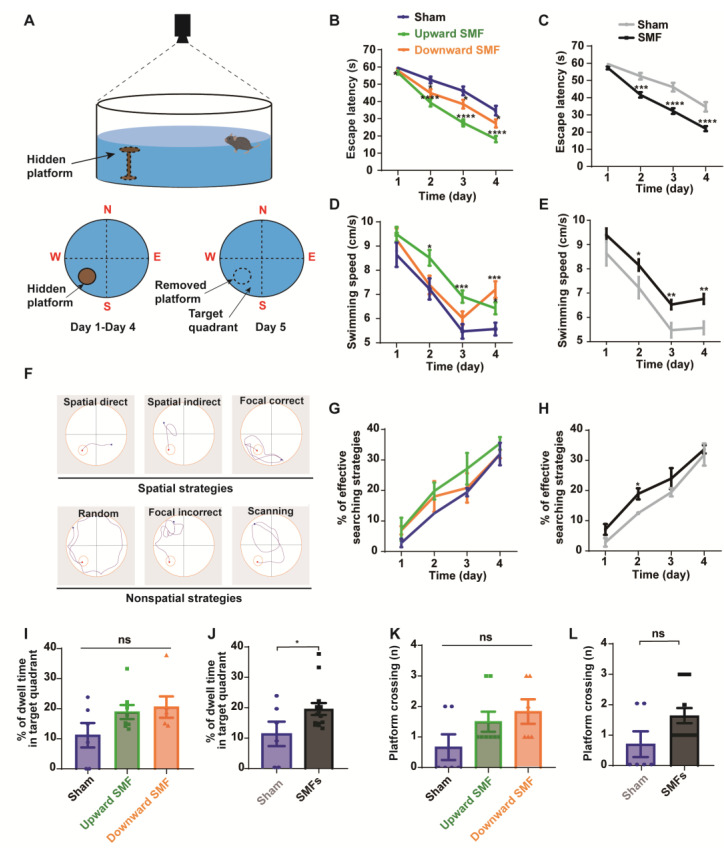
Morris water maze test shows that SMFs could improve spatial learning ability and spatial memory of aged mice. (**A**) Illustration of Morris water maze device, (**B**,**C**) escape latency during the spatial acquisition training days, (**D**,**E**) swimming speed during the spatial acquisition training days, (**F**) different types of search strategies during the spatial acquisition training days, (**G**,**H**) the percentage of effective searching strategies during the spatial acquisition training days, (**I**,**J**) the percentage of dwell time in the target quadrant during the probe trial, (**K**,**L**) platform crossing during the probe trial were recorded and analyzed in a blinded way. Mice in upward or downward SMF exposure groups were analyzed separately (**B**,**D**,**G**,**I**,**K**) or grouped together as SMF group (**C**,**E**,**H**,**J**,**L**). n = 6–8 mice for the sham, upward SMF or downward SMF group. Values were expressed as means ± SEM. * *p* < 0.05, ** *p* < 0.01, *** *p* < 0.001, and **** *p* < 0.0001 by Student’s *t*-test. ns, no significance.

To assess the effects of an exposure to SMFs on the spatial learning and memory ability, the Morris water maze test was conducted on 84-week-old mice (Figure 4). Their spatial learning ability was measured by calculating the latency to access the hidden platform during the spatial acquisition training days, when the mice were guided to find the platform on day 1 to day 4. The spatial memory could be assessed by measuring the percent of the dwell time in the target quadrant and platform crossings during the final probe trial on day 5, when the hidden platform was removed. 

During the 4-day training, we found that the escape latency decreased significantly in the upward and downward SMF group compared with that of the sham group, indicating an improved spatial learning ability. The escape latency in the upward SMF group was reduced by 4.70% (*p* < 0.05 on day 1), 25.32% (*p* < 0.0001 on day 2), 40.21% (*p* < 0.0001 on day 3), and 47.63% (*p* < 0.0001 on day 4), respectively. The escape latency in the downward SMF group was reduced by 2.79% (*p* > 0.05 on day 1), 14.64% (*p* < 0.05 on day 2), 16.67% (*p* < 0.05 on day 3), and 21.62% (*p* < 0.05 on day 4), respectively, compared to the sham group (Figure 4B). In general, the escape latency in the SMF group reduced by 3.88% (*p* > 0.05 on day 1), 20.74% (*p* < 0.001 on day 2), 30.13% (*p* < 0.0001 on day 3), and 36.49% (*p* < 0.0001 on day 4), respectively, compared to the sham group (Figure 4C). We also noticed that the swimming speed was faster in the SMF group than the sham group during the 4-day training (Figure 4D,E), indicating an improved physical state of the SMF-treated mice, which is consistent with the open field test. 

The mice searching path can be analyzed for the frequency of their spatial strategies, which are more effective than the non-spatial strategies (Figure 4F) [26]. We found that the effective spatial strategies during the 4-day acquisition training were more frequently used by the SMF-exposed mice than the sham group mice when they looked for the hidden platform (Figure 4G,H). For example, the percent of effective searching strategies on the second day in the SMF treatment group was increased by 51.33% (*p* < 0.05) compared to the sham group (Figure 4H), indicating an improved spatial learning ability. 

To compare their spatial memory ability, the platform was removed on day 5. We found that the percent of dwell time in the target quadrant increased in the SMF groups compared with the sham group (*p* < 0.05) (Figure 4J), indicating an improved spatial memory ability in SMF-treated mice. The platform crossing frequency was also increased in the SMF groups, but there was no statistical significance (Figure 4L). In general, the mice in the SMFs group had an improved spatial memory ability compared with the sham group (Figure 4I–L).

### 3.4. Oxidative Stress Was Decreased by SMFs in Mice Brain

Since oxidative stress plays a crucial role in the development of ageing, and magnetic fields have been shown to affect the reactive oxygen species (ROS) levels [17,18], we examined the oxidative stress in the mice to evaluate whether the mechanism of an SMF-induced mice physiology improvement is associated with the oxidative stress levels (Figure S5). We found that the total antioxidant capacity of the mice brain was generally increased by an SMF treatment (*p* = 0.05, for downward and upward SMF groups combined together), especially by the downward SMF treatment (*p* < 0.05) (Figure S5A,B). The SOD activity of the mice brain in the SMF group also had a higher tendency, but this was not statistically significant (Figure S5C,D). Similarly, compared with the sham group, the LPO and MDA content of the mice brain in the SMF group had a decreased tendency, but this was not statistically significant (Figure S5E–H). These results indicated that SMFs might improve the physiological state of mice by decreasing the oxidative stress levels. 

### 3.5. SMFs Protected the PC12 Cells from Senescence by Suppressing Oxidative Stress In Vitro

**Figure 5:**
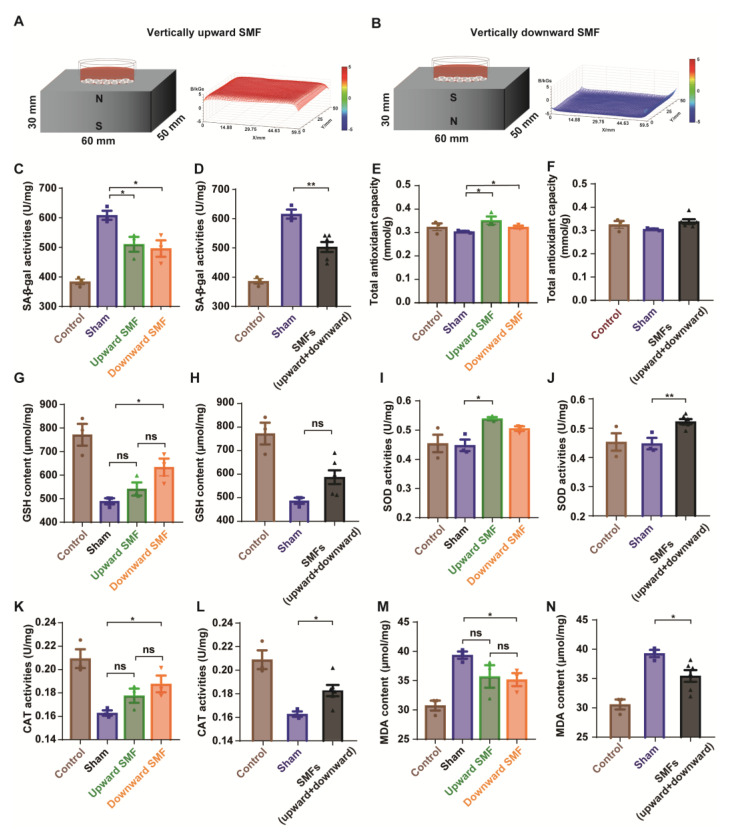
SMFs could protect the PC12 cells from D-gal-induced senescence by reducing oxidative stress. (**A**,**B**) Experimental setup. The distribution of SMFs at 1 mm above the magnets were measured by a magnet analyzer; (**C**,**D**) senescence-associated β-galactosidase activity assay; (**E**,**F**) total antioxidant capacity in PC12 cells; (**G**,**H**) GSH content in PC12 cells; (**I**,**J**) SOD activity in PC12 cells; (**K**,**L**) CAT activity in PC12 cells; (**M**,**N**) MDA content in PC12 cells; cell in vertically upward or downward SMF exposure groups were analyzed separately (**C**,**E**,**G**,**I**,**K**,**M**) or grouped together as SMF group (**D**,**F**,**H**,**J**,**L**,**N**). The experiments have been repeated for 3 times and the values are means ± SEM. * *p* < 0.05, ** *p* < 0.01 by Student’s *t*-test. ns, no significance.

To further explore the effects of SMFs on the senescent cells, we used rat pheochromocytoma PC12 cells induced by D-gal in vitro. The cells were placed on the top of the magnetized or unmagnetized NdFeB magnets where they were exposed to the sham control, or ~0.1 T–0.5 T SMFs (Figure 5A,B). As expected, the cell viability was dose-dependently decreased by the D-gal treatment, confirming the toxicity of D-gal (Figure S6). The SA-β-gal activity was considered as an indicator of the cellular senescence [27], which was used to assess the D-gal-induced cellular senescence. Our results show that the SA-β-gal activity of the PC12 cells treated with 20 mg/mL of D-gal for 48 h was significantly decreased by SMFs (Figure 5C,D and Figure S7), indicating that SMFs could protect the PC12 cells from a D-gal-induced senescence.

To further investigate the mechanisms, multiple oxidative stress biomarkers were measured. Consistent with the mice brain tissue results, the antioxidative stress effects of SMFs on D-gal-induced senescent PC12 cells were also observed, including the total antioxidant capacity (T-AOC), superoxide dismutase (SOD), and catalase (CAT) activities, as well as glutathione (GSH), lipid hydroperoxide (LPO), nitric oxide (NO), and malonaldehyde (MDA) contents (Figure 5E–N and Figure S8). 

## 4. Discussion

There are many people using magnetotherapy products by themselves, without the instruction of physicians [28], and most of them are older people. However, moderate to high SMFs are known to generate various bioeffects on a living organism, and the exact effects of a long-term SMF exposure are not particularly clear. Therefore, the purpose of this study was originally designed to detect the potential harmful effects of a long-term SMF exposure on older mice. We started to apply SMF when the healthy C57BL/6 mice were 1 year old, which was the equivalent of ~40-year-old people, until the mice died naturally. Surprisingly, our data show that the 1.7-year continuous long-term SMF exposure did not have any detectable harmful effects on these mice. On the contrary, the moderate SMF exposure can even improve the physiological state of naturally aged mice and prolong their survival by reducing the oxidative stress, which was confirmed by PC12 cellular assays.

By performing behavior experiments, it is clear that the untreated mice had a decreased locomotive and exploratory activity at the later stage, which is the natural consequence of ageing. However, the SMF treatment maintained the locomotive and exploratory activity at a younger mice level. For example, there was no obvious reduction in the locomotive and exploratory activity of the SMF-treated mice at 107 weeks old, compared to 56-week-old mice. In contrast, in the control group without an SMF treatment, the locomotive and exploratory activity of the mice who were 107 weeks old was decreased by 70.95% compared to the 56-week-old mice.

Oxidants are produced by the normal intracellular metabolism of mitochondria, peroxisomes and various cytoplasmic enzymatic systems, and an increased ROS could be harmful, leading to cell death or accelerated ageing and age-related diseases [29,30]. Multiple studies have reported that oxidative stress is directly correlated with the lifespan and healthspan [27,29,31,32,33,34,35]. For example, Dominika et al. found that moderate and short-term calorie restriction could decrease the body weight and improve the cardiovascular and metabolic parameters in obese patients by reducing the oxidative stress levels [35]. SMFs have been reported to improve the healthspan of mice [19]. Bellossi et al. [19] exposed female spontaneous lymphocytic leukemia mice with uniform 600 or 800 mT SMFs for 2 h per day, 5 days per week for one year and found that 50% of the mice had a more prolonged survival [19]. More importantly, a 10 mT SMF has been recently reported to promote the nematode longevity through cytochrome P450 enzymes [15]. However, Huang et al. found that 200 mT of SMF could reduce the nematode longevity [36] and Lee et al. exposed nematode with 150 mT or 200 mT of SMF for 4 or 8 days and found that SMFs could decline the crawling speed and mobility of nematode [37]. The effects of SMFs on the ageing of healthy mice has bever been reported, especially the effects of a long-term exposure. 

Moreover, many studies have reported that SMFs could influence the oxidant stress levels [17], which was because the production of free radicals could be influenced by the radical-pair recombination changes induced by SMFs [38,39,40]. For example, Wang et al. [18] found that the ROS level could be affected by moderate SMF in MCF7, RPE1, C6, U251, HepG2, GIST-T1, and EJ1 cells, but not CHO, 293 T, and PC12 cells. However, the exact consequences of the ROS levels and the mechanisms that caused the different effects after the SMF exposure remain unclear [17]. In addition, Serrano et al. [41] found that the production of SOD was increased for S. obliquus and N. gaditana after the SMF exposure. These results are consistent with the view of Ferrada et al. [42], where the enzymatic activity of the SOD was increased after an SMF exposure in N. gaditana. Furthermore, SMFs also have been shown to be able to affect some enzymatic activity [43]. However, our work here is the first study that have investigated the effect of SMFs on ageing mice, and to show that SMFs could reduce the oxidative stress to delay ageing.

The major limitation of our study is the sample size. Our magnet plates are made from big chunks of custom-made Neodymium magnets (25 cm × 16 cm × 4.5 cm), which provide a relatively high magnetic flux than most other permanent magnetic devices in the literature and need to be placed far apart from each other to avoid an attraction between them. Moreover, we placed the whole mice cage on the magnet plates for ~2 years. Therefore, the total number of experimental animals is toward the lower end. Second, limited by the number of experimental animals and our desire to observe their natural ageing and death, the oxidative stress indicators and pathological sections were not comprehensively investigated here. Alternatively, we used cellular assays to evaluate the phenomenon we observed in the animal experiments. Using a D-gal-induced PC12 cell senescence model to simulate the process of neuronal cell senescence, we found that the SA-β-gal activity was decreased by SMFs, suggesting that the SMFs can suppress the D-gal-induced PC12 cell senescence. The SMFs also regulated an unbalanced antioxidant system formed in the D-gal-induced senescent PC12 cells, as confirmed by the change in the T-AOC, SOD, and CAT activity, as well as the GSH, LPO, NO, and MDA content. These results confirmed that the moderate intensity SMFs we used could reduce the oxidative stress in the nerve cells. Moreover, although we have compared SMFs of two different directions and, in fact, we have recently written a book chapter [44] about the differential bioeffects of SMFs of different directions, it is still hard to draw unambiguous conclusions or rules yet. The mechanism is still an open question that is also one of the focuses of our future work, which we believe can also clarify some of the inconsistencies in the literature about the bioeffects of SMFs. 

Lastly, it should also be mentioned that we treated the mice with SMFs for 16 h a day for 5 weeks and for 24 h thereafter. Our original plan was to expose the mice to SMFs for 16 h a day throughout the whole experiment because most previously reported studies have only exposed mice to SMFs for a few hours a day. Since we did not find any abnormalities of the mice, we wanted to see what could happen if we exposed them continuously to the SMFs. In another word, we wanted to explore the potential harmful effects of such a prolonged exposure. However, apparently, we did not find any, at least from this study. Although, we do not exclude the possibility that more thorough examinations might reveal some unidentified effects, which is also a direction for future investigations.

## 5. Conclusions

Here, we studied the effect of a ~1.7-year moderate, continuous exposure to SMFs (70-220 mT head-to-toe) on the physiological state of naturally aged mice and found that their lifespan and healthspan were both improved. This is the only experimental study that we know of which has investigated the long-term effects of SMFs for longer than a year. Our animal study and cellular assays both show that these moderate SMFs of a few hundred mT could effectively reduce the cellular oxidative stress, which consequently improves the exploratory and locomotive activities, as well as the spatial learning and memory of mice. Therefore, our work reveals a potential for SMFs to be developed into an adjuvant physical method to reduce ageing- and oxidative stress-related health risks in the future.

## Figures and Tables

**Table 1:** Effects of static magnetic field exposure on complete blood count indicators in mice. Statistical analysis was performed and no significant differences were found.

	Sham Mean ± SD (Range)	Upward Mean ± SD (Range)	Downward Mean ± SD (Range)
Red blood cell (10^12^/L)	8.22 ± 0.22	8.29 ± 0.04	8.13±0.39
Hematocrit(%)	39.60 ± 1.04	40.33 ± 0.75	40.07 ± 1.38
Hemoglobin (g/L)	11.73 ± 0.37	11.93 ± 0.33	11.77 ± 0.63
Platelet (10^9^/L)	1737.67 ± 427.58	1833 ± 196.03	1675 ± 511.32
Neutrophil (%)	22.03 ± 8.72	13.87 ± 1.88	13.70 ± 1.49
Eosinophil (%)	3.67 ± 3.42	1.73 ± 0.37	1.07 ± 0.40
Monocyte (%)	12.73 ± 1.93	12.20 ± 1.37	12.73 ± 2.18
Lymphocyte (%)	61.33 ± 12.96	71.03 ± 2.50	72.30 ± 3.12
Mean corpuscular volume (fL)	48.20 ± 1.62	48.63 ± 0.68	49.33 ± 0.74
Mean platelet volume (fL)	7.77 ± 0.52	7.70 ± 0.36	7.23 ± 0.25
Plateletcrit (fL)	1.32 ± 0.26	1.55 ± 0.09	1.51 ± 0.32
Mean corpuscular hemoglobin (pg)	14.30 ± 0.57	14.37 ± 0.34	14.50 ± 0.51
Mean corpuscular hemoglobin concentration (g/L)	29.67 ± 0.19	29.60 ± 0.54	29.33 ± 0.91
Platelet distribution width (fL)	8.50 ± 0.64	8.60 ± 0.65	7.87 ± 0.25

## Data Availability

Data is contained within the article and supplementary material.

## Supplementary Materials

The following supporting information can be downloaded at: https://www.mdpi.com/article/10.3390/antiox12010108/s1, Figure S1. Body weight, food, and water consumption of mice treated with sham or SMFs. Figure S2. Complete blood count of naturally aged mice. Figure S3. Time in the open arms, total travelled distance, and total average velocity of mice treated with sham or SMFs in elevated plus maze test. Figure S4. Representative heat plots of mice movement in the open field test. Figure S5. SMFs have an ameliorative effect on oxidative stress in the brain of aged mice. Figure S6. The relative cell viability was dose-dependently decreased by D-gal treatment in PC12 cells. Figure S7. Senescence-associated β-galactosidase staining of senescent PC12 cells treated by SMFs. Figure S8. The NO content of senescent PC12 cells treated by SMFs.
