# Supplementary material for: Life on Magnet: Long-Term Exposure of Moderate Static Magnetic Fields on the Lifespan and Healthspan of Mice
Source: Antioxidants (Basel). 2022 Dec 31;12(1):108. doi: 10.3390/antiox12010108 (PMC9854752; doi:10.3390/antiox12010108)
Supplement: Supplementary file 1 [file antioxidants-12-00108-s001.zip › antioxidants-2085648-supplementary.pdf]

Supplementary Materials

Life on magnet: long-term exposure of moderate static magnetic fields on the lifespan and healthspan of mice

Yixiang Fan[1,2], Xin Yu[1,2], Biao Yu[1], Xinmiao Ji[1], Xiaofei Tian[3], Chao Song[1], Xin Zhang[1,2,3,4*]

[1] High Magnetic Field Laboratory, Hefei Institutes of Physical Science, Chinese Academy of Sciences, Hefei 230031, China

[2] Science Island Branch of Graduate School, University of Science and Technology of China, Hefei 230036, China

[3] Institutes of Physical Science and Information Technology, Anhui University, Hefei 230601, China

[4]International Magnetobiology Frontier Research Center, Science Island, Hefei 230036, China

*Correspondence: xinzhang@hmfl.ac.cn

Supplementary Figures.

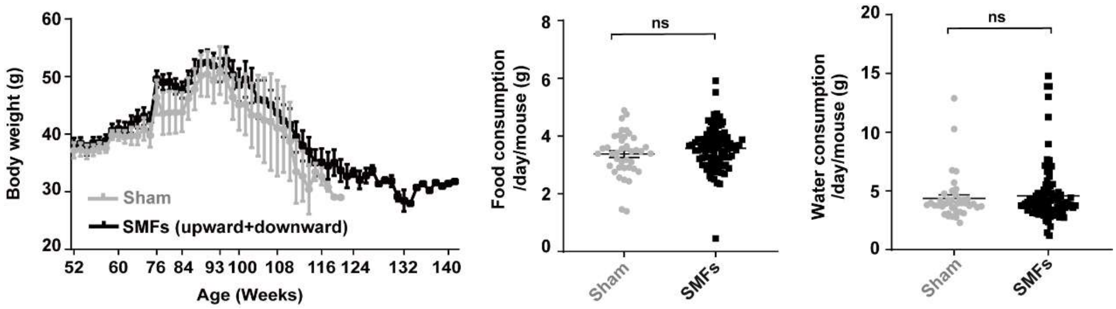

Figure S1. Body weight, food and water consumption of mice treated with sham or SMFs. The mice in the vertically upward and downward SMFs are pooled together for data analysis. n=8 mice for the sham, upward SMF or downward SMF group in the beginning of the experiment. Values were expressed as means ± SEM. ns, no significance.

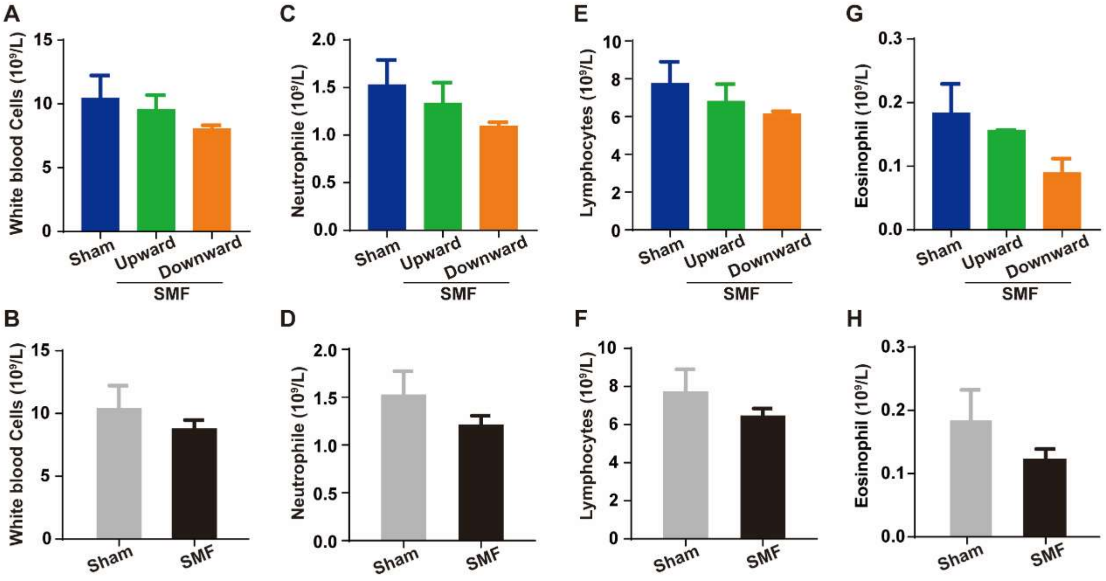

Figure S2. Complete blood count of naturally aged mice. (**A**, **B**) White blood cells; (**C**, **D**) Neutrophile; (**E**, **F**) Lymphocytes; (**G**, **H**) Eosinophil. Mice in upward or downward SMF exposure groups were analyzed separately (**A**, **C**, **E**, **G**) or grouped together as SMF group (**B**, **D**, **F**, **H**). n = 3 mice for the sham, upward SMF or downward SMF group. Values were expressed as means ± SEM.

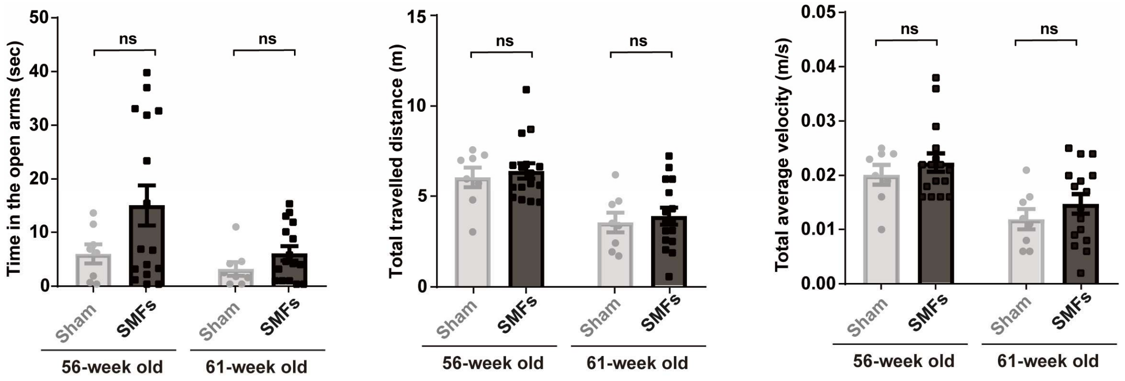

Figure S3. Time in the open arms, total travelled distance and total average velocity of mice treated with sham or SMFs in elevated plus maze test. The mice in the vertically upward and downward SMFs are pooled together for data analysis. n=8 mice for the sham, upward SMF or downward SMF group. Values were expressed as means ± SEM. ns, no significance.

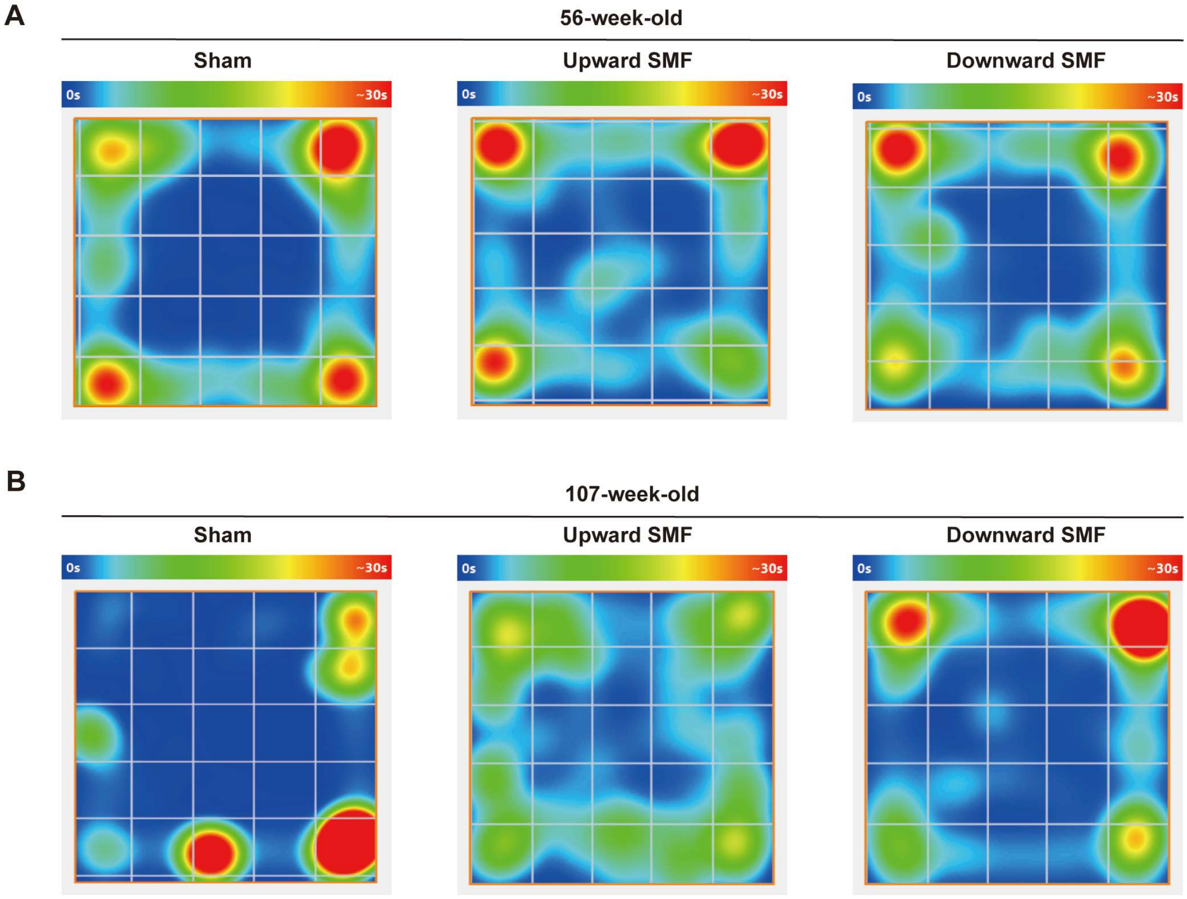

Figure S4. Representative heat plots of mice movement in the open field test. (**A**) Heat plots of mice movement in the open field test at 56-week-old; (**B**) Heat plots of mice movement in the open field test at 107-week-old. n=8 mice for the sham, upward SMF or downward SMF group in the beginning of the experiment.

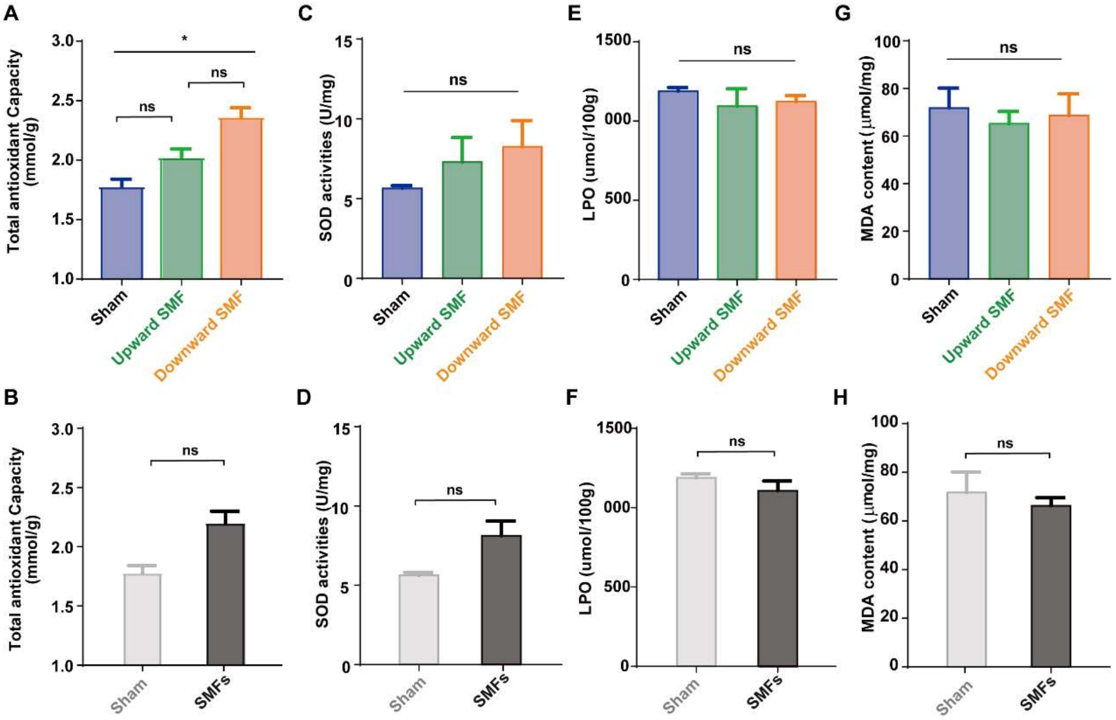

Figure S5. SMFs have an ameliorative effect on oxidative stress in the brain of aged mice. (**A**, **B**) Total antioxidant capacity in the aged mice brain; (**C**, **D**) SOD activity in the aged mice brain; (**E**, **F**) LPO content in the

mice brain; (**G**, **H**) MDA content in the mice brain. Brain tissue in vertically upward or downward SMF exposure groups were analyzed separately (**A**, **C**, **E**, **G**), or grouped together as SMF group (**B**, **D**, **F**, **H**). Values were expressed as means ± SEM. n=2-4 mice for the sham, upward SMF or downward SMF group. * $p < 0.05$ by Student *t*-test. ns, no significance.

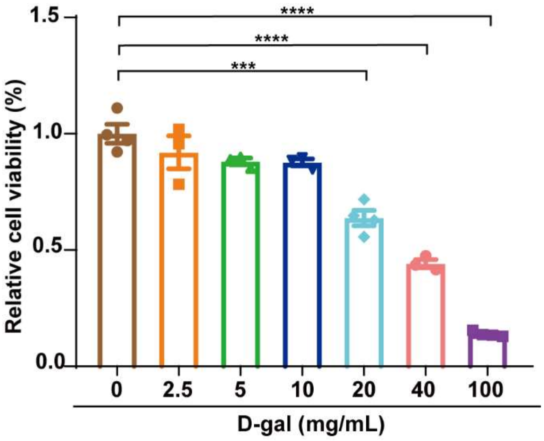

Figure S6. The relative cell viability was dose-dependently decreased by D-gal treatment in PC12 cells. Values were expressed as means ± SEM. The experiments have been repeated for 3 times or 4 times. Values were expressed as means ± SEM. *** $p < 0.001$ and **** $p < 0.0001$ by Student *t*-test.

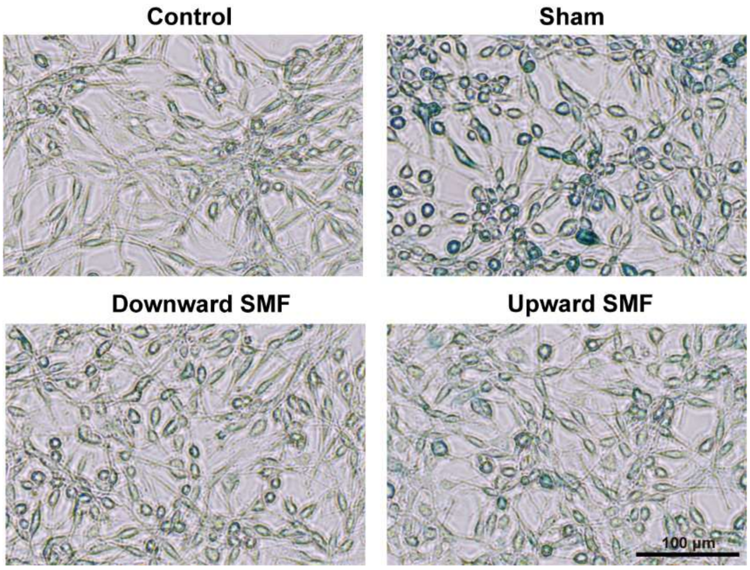

Figure S7. Senescence-associated β-galactosidase staining of senescent PC12 cells treated by SMFs. Scale bar: 100 μm. The experiments have been repeated for 3 times and representative images are shown.

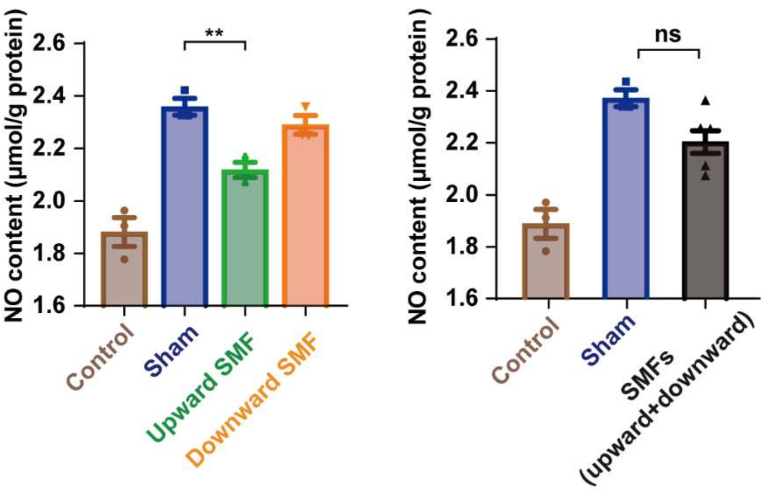

Figure S8. The NO content of senescent PC12 cells treated by SMFs. PC12 cells in the vertically upward and downward SMF group were analyzed separately or grouped together as SMFs group. The experiments have been repeated for 3 times and the values are means ± SEM. ** $p < 0.01$ by Student's *t*-tests. ns, no significance.
